# The Association Between Walking Speed and Bone Turnover Markers in Older Adults

**DOI:** 10.7759/cureus.18019

**Published:** 2021-09-16

**Authors:** Carlos H Orces

**Affiliations:** 1 Internal Medicine/Rheumatology, Laredo Medical Center, Laredo, USA

**Keywords:** epidemiology, exercise, osteoporosis, older adults, walking speed, bone turnover markers

## Abstract

Background

Previous research conducted among institutionalized older adults has reported increased bone turnover markers (BTMs) in those subjects with mobility limitation. However, the association between walking speed and bone metabolism has not been well described in community-dwelling older adults.

Methods

The National Health and Nutrition Examination Survey 1999-2000 and 2001-2002 cycles were used to determine the association between the 20-feet walking speed test and bone alkaline phosphatase (BAP) and the cross-linked N-telopeptides of type I bone collagen (NTx). Sex-specific general linear models adjusted for potential confounders were assembled to examine the independent association between the time to complete the walking speed test grouped into quartiles and the logarithmic transformations of BAP and NTx levels.

Results

Of 2,521 older adults, 25.8% were defined as having mobility limitation. In both genders, BTM levels progressively increased as the time to complete the walking test also increase. Indeed, women and men who completed the walking speed test in the worst time quartile had on average 6% and 2.8% higher NTx levels than their counterparts who completed the test in the best time quartile, respectively. Likewise, BAP levels also progressively increased across walking speed time quartiles, but to a lesser degree. Notably, NTx levels were 4.7% and 2.6% higher in women and men with mobility limitation than those without, respectively. In contrast, BAP levels did not significantly differ in older adults regardless of their mobility limitation status.

Conclusions

Community-dwelling older adults with slower walking speeds and mobility limitation consistently had evidence of increased bone resorption. Thus, the present findings indicate that older adults with mobility limitation should be considered at risk of osteoporosis.

## Introduction

Osteoporosis is defined as a progressive systemic skeletal disease characterized by low bone mass and microarchitectural deterioration of bone tissue, with a consequent increase in bone fragility and susceptibility to fracture [1]. Bone turnover markers (BTMs) reflect the cellular activities of bone formation and resorption, and have been used to assess the efficacy of physical activity on bone remodeling [2]. Recently, the results of a systematic review of randomized controlled trials among middle-aged and older adults demonstrated that acute aerobic, resistance, or impact exercise is an effective intervention in modifying BTMs, which varies according to the exercise intensity, age, and sex [3]. In contrast, research conducted among institutionalized frail older adults reported that BTMs increased with age in both sexes and were higher in women [4-6]. Moreover, older adults with poor mobility had significantly higher markers of bone resorption than their physically active counterparts [5,6]. Notably, Chen et al. in a large study conducted in nursing home and intermediate care facilities residents also demonstrated that poor mobility was significantly associated with increased markers of bone formation [4].

Relatively a few population-based studies have explored the relationship between mobility limitation and BTM’s in older adults. Previously, among Japanese aged 40 years and older, only women who self-reported walking difficulty had significantly higher levels of bone resorption than their counterparts who did not. In addition, the effect of mobility limitation on markers of bone formation was not evaluated [7]. Thus, the present study aimed to examine the effect of walking speed and mobility limitation on BTMs in adults aged 60 years and older. It was hypothesized that slow walkers and those with mobility limitation will have higher BTMs levels. 

## Materials and methods

Study sample 

The present analysis was based on data from the continuous National Health and Nutrition Examination Survey (NHANES) 1999-2000 and 2001-2002 cycles. The NHANES is designed to assess the health and nutritional status of adults and children in the United States (U.S). A complex, multistage probability sampling design was used to select a sample representative of the civilian noninstitutionalized household population of the U.S. The NHANES protocol was approved by the National Center for Health Statistics Research Ethics Review Board (study protocol # 98-12). Documented signed consent was obtained from all participants [8].

Covariates

The demographics characteristics of the participants were self-reported. Subjects were considered to drink alcohol if they reported at least 12 drinks of any type of alcoholic beverage in a year? Smoking status was classified as never, former, or current smokers. Muscle-strengthening activities and its frequency were assessed by asking the participants “Over the past 30 days, did you do any physical activities specifically designed to strengthen your muscles such as lifting weights, push-ups or sit-ups?" Moreover, participants reported the time spend on a typical day sitting and watching TV or videos or using a computer outside work over the past 30 days. In the medical examination center, the body measurement assessment was performed and the BMI was calculated in kg/m^2^

In the dietary interview component, participants reported the total amount of calcium consumed during the 24-hour period prior to the interview. Diagnosis of osteoporosis was evaluated by asking participants “Has a doctor ever told you that you had osteoporosis, sometimes called thin or brittle bones” Similarly, medications prescribed for osteoporosis in the previous 30 days (alendronate, ibandronate, risedronate, calcitonin, risedronate, teriparatide, denosumab) were reported according to the therapeutic classification scheme of Cerner Multum’s Lexicon.

Walking speed test

Participants walked a 20 feet-long test track, which was timed using a hand-held stopwatch. For this test, adhesive tape strips on the floor indicated the start and stop points for walking. Walking speed in feet/second was transformed to meters/second (1 foot = 0.3048 meters) and subjects who completed the test with a gait speed < 0.8 meters/second were defined as having mobility limitation [9]. 

Bone turnover markers

The laboratory methodology of the bone alkaline phosphatase (BAP) and the cross-linked N-telopeptides of type I bone collagen (NTx) laboratory is described at https://wwwn.cdc.gov/Nchs/Nhanes/2001-2002/LAB11.htm. Briefly, the NHANES 1999-2000 BAP (ug/L) was measured using the Ostase assay and the Hybritech Ostase assay and the Beckman Access Ostase assay during the cycle 2001-2002. The Osteomark, was used for the quantitative measurement of the NTx (nmol BCE) in urine and then was measured with the Vitros Eci instrument in 2002. As a result of using different assay methods, the NHANES adjusted the BAP and NTx levels for the cycle 2001-2002. 

Statistics analysis

SPSS Complex Sample software, V.25 (SPSS Inc, Chicago, IL, USA) was used to incorporate constructed weights and obtain unbiased national estimates for the combined survey cycles. The characteristics of participants were stratified according to mobility limitation status. The Chi-squared and t-test were used to compare categorical and continuous variables, respectively. Logarithmic transformations (Ln) of BAP and NTx levels were then calculated to achieve normal distributions. For this analysis, sex-specific general linear models were assembled to assess the independent association between the time to complete a 20 feet-long test track stratified into quartiles and LnBTMs levels, using the first quartile (fast walkers) as the reference category. These models were adjusted for demographic variables and potential confounders previously reported to increase the risk of osteoporosis [10]. Similarly, BTMs levels were compared between participants with and without mobility limitation. A p-value < 0.05 was considered statistically significant. Of 3,706 participants aged 60 years and older, those with missing data on walking speed, BMI, calcium intake, and BTMs were excluded, leaving a final sample size of 2,521 subjects. 

## Results

The mean age of the participants was 70.9 (SD 7.6) years and women accounted for 55.7% of the study sample. Overall, 25.8% of older adults were defined as having mobility limitation, representing an estimated 9.2 million older U.S. adults during the study period. As shown in Table 1, participants with mobility limitation were more likely to be older, women, non-Hispanic black, higher BMI, a sedentary lifestyle, lower calcium intake, and self-reported a diagnosis of osteoporosis. 

**Table 1:** Characteristics of participants according to mobility limitation status. BMI: body mass index; LnBAP: logarithmic transformations of bone-specific alkaline phosphatase; LnNTX: logarithmic transformations of N-telopeptides of type I bone collagen.

	Mobility limited (n= 763)		Non-limited (n= 1,758)	p-value
Age (years), mean		74.5	68.9	<0.0001
Sex, %				<0.0001
Male		18.5	81.5	
Female		31.5	68.5	
Race/ethnicity, %				<0.0001
Hispanic		33.1	66.9	
Non-Hispanic White		21.8	76.2	
Non-Hispanic Black		40.6	59.4	
Others		27.8	72.2	
BMI (kg/m^2^)		28.9	27.9	<0.0001
Alcohol use, %				<0.0001
Yes		20.5	79.5	
No		33.6	66.4	
Smoking, %				<0.0001
Never		29.5	70.5	
Former		22.0	78.0	
Current		23.4	76.6	
Strengthening exercise, %				<0.0001
Yes		17.0	83.0	
No		24.9	75.1	
TV, video, or computer use, %				<0.0001
< 1 h		22.6	77.4	
1-2 h		20.6	79.4	
≥ 3 h		29.8	70.2	
Calcium intake (mg), mean		665.8	752.3	<0.0001
Osteoporosis history, %				<0.0001
Yes		40.2	59.8	
No		23.6	76.4	
Osteoporosis treatment, %				0.143
Yes		23.5	76.5	
No		25.9	74.1	
Ln BAP (ug/L), mean		2.7	2.6	<0.0001
Ln NTx (nmol BCE), mean		5.6	5.4	<0.0001

Table 2 shows BTMs levels stratified according to quartiles of time (seconds) needed to complete a 20-feet walk.

**Table 2:** Quartiles of time to complete the 20-feet walking test and bone turnover markers levels in older adults. *p < 0.05 compared with Q1 as the reference category. Model 1: adjusted for age, race/ethnicity, and BMI. Model 2: adjusted for model 1, and alcohol use, smoking status, sedentary lifestyle, strengthen exercise, calcium intake, history of osteoporosis, and anti-osteoporosis medications. LnBAP: logarithmic transformations of bone-specific alkaline phosphatase; LnNTx: logarithmic transformations of N-telopeptides of type I bone collagen.

Women	Q1 (1.64-5.72 sec)	Q2 (5.73-6.78 sec)	Q3 (6.79-8.31 sec)	Q4 (≥ 8.32 sec)
LnBAP (ug/L)				
Model 1	2.61	2.67	2.69	2.76*
Model 2	2.62	2.67	2.67	2.74*
LnNTx (nmol BCE)				
Model 1	5.23	5.26	5.32	5.59*
Model 2	5.26	5.24	5.28	5.58*
Men	Q1 (3.69-5.52 sec)	Q2 (5.53-6.34 sec)	Q3 (6.35-7.65 sec)	Q4 (≥ 7.66 sec)
LnBAP (ug/L)				
Model 1	2.61	2.65	2.70	2.72*
Model 2	2.61	2.66	2.69*	2.69*
LnNTx (nmol BCE)				
Model 1	5.54	5.61	5.56	5.73*
Model 2	5.55	5.60	5.56	5.71*

In general, BTM levels in both genders progressively increased as the time to complete the walking test also increase. Notably, BTMs levels were significantly higher among older adults who completed the waking test in the worst score than those grouped in the best time quartile. In addition, this difference was more marked in women. Indeed, after adjusting for potential confounders, women who completed the walking test in ≥ 8.32 sec had on average 5.7% and 4.3% higher NTx and BAP levels than those who completed the test in ≤ 5.72 sec, respectively. 

As shown in Table 3, overall, 31.5% of women and 18.5% of men were defined as having mobility limitation. Notably, after controlling for potential confounders, NTx levels were 4.7% and 2.6% higher in women and men with mobility limitation than those without, respectively. In contrast, BAP levels were comparable in older adults regarding their mobility limitation status. 

**Table 3:** Bone turnover markers levels difference according to mobility limitation status in older. Model 1: adjusted for age, race/ethnicity, and BMI. Model 2: adjusted for model 1, and alcohol use, smoking status, sedentary lifestyle, strengthen exercise, calcium intake, history of osteoporosis, and anti-osteoporosis medications. LnBAP: logarithmic transformations of bone-specific alkaline phosphatase; LnNTx: logarithmic transformations of N-telopeptides of type I bone collagen.

Women	Limited (n = 440)	Non-limited (n = 817)	Difference	p-value
LnBAP (ug/L)				
Model 1	2.73	2.65	0.079	<0.05
Model 2	2.71	2.65	0.056	0.107
LnNTx (nmol BCE)				
Model 1	5.52	5.25	0.265	<0.0001
Model 2	5.50	5.25	0.253	<0.0001
Men	Limited (n = 323)	Non-limited (n = 941)	Difference	p-value
LnBAP (ug/L)				
Model 1	2.72	2.65	0.069	<0.05
Model 2	2.68	2.65	0.033	0.326
LnNTx (nmol BCE)				
Model 1	5.73	5.57	0.168	<0.05
Model 2	5.72	5.57	0.155	<0.05

**Figure 1:**
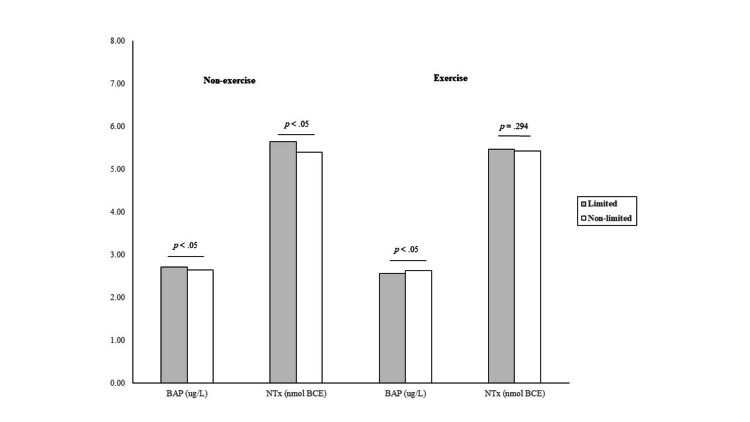
Bone turnover markers according to mobility limitation status and muscle-strengthening exercise. BAP: bone alkaline phosphatase; NTx: N-telopeptides of type I collagen.

As shown in Figure 1, in a subgroup analysis stratified according to self-reported muscle strengthening exercise and mobility limitation status, participants who were physically inactive and mobility limited had 2.5% and 4.4% higher BAP and NTx levels than their counterparts without walking limitation, respectively. In contrast, comparable NTx levels were seen between older adults with and without mobility limitation who reported muscle-strengthening activities in the previous month. 

## Discussion

The results of this cross-sectional analysis indicate that older adults with slower walking speeds had consistently increased bone turnover markers. Indeed, women and men who completed the walking speed in the worst time quartile had on average 6% and 2.8% higher NTx levels than their counterparts who completed the test in the best time quartile, respectively. Similarly, increased BAP levels were seen as the time to complete the walking test also increase, but to a lesser degree. Moreover, older adults defined with mobility limitation had significantly higher NTx levels than those without. In contrast, BAP levels did not significantly differ in older adults regardless of their mobility limitation status. 

The present findings are consistent with those reported among frail and institutionalized older adults in which residents with poor mobility had significantly higher markers of bone resorption than their physically active counterparts [4-6]. Moreover, previous randomized controlled trials conducted in postmenopausal women demonstrated that daily walking activity significantly lower deoxypyridinoline levels, a marker of osteoclastic activity, compared with those physically inactive [11,12]. Moreover, a small study in postmenopausal women with osteopenia and/or osteoporosis reported that NTx levels decreased by 25% in the walking exercise group after the third month, and this reduction was sustained until the end of the study [13]. Thus, the present results suggest that mobility limitation in older adults significantly increases bone resorption, which has been described as a major determinant of osteoporosis and risk of fragility fractures [14,15]. Likewise, prospective cohort studies in older adults have demonstrated that walking speed and the greatest declines in walking speed over time were associated with increased risk of hip and fragility fractures [16,17].

Of relevance, a recent study conducted among postmenopausal women with low bone mass demonstrated that a single session of resistance exercise decreased C-telopeptides of type I bone collagen by 8.9% from baseline levels, which is in agreement with the present results. [18]. Indeed, among participants with sedentary lifestyle and mobility limitation had on average 4.6% higher NTx levels than their counterparts without limitation. In contrast, comparable bone resorption levels were seen among older adults who performed resistance exercise in the previous 30 days regardless their mobility limitation status. Beavers et al. also reported that among overweight and obese older adults, resistance training rather than weight-bearing aerobic training during weight loss may attenuate the decrease in hip and femoral neck bone mineral density [19]. Similarly, a systematic review of the effects of different types of training programs on bone mass in older adults reported that walking provides a modest increase in the loads on the skeleton above gravity. However, strengthening exercise seems to be a powerful stimulus to improve and maintain bone mass during the ageing process [20]. 

Although it is well established that older adults with mobility limitation are at increased risk of falls, hip fracture, institutionalization, and even death [21], the mechanism for which mobility limitation is particularly correlated with increased bone resorption has not been completely elucidated. Nevertheless, a cross-sectional analysis of pooled data from 18,765 older adults reported that decreased grip strength, with and without adjustments for body size and composition consistently discriminated individuals with mobility limitation, suggesting a strong association between both conditions [22]. Luo et al. also demonstrated that grip strength was positively associated with femoral neck and total lumbar spine BMD in men, premenopausal, and postmenopausal women. In that latter study, the authors postulated that stronger muscles exert greater stretch on bones. Moreover, muscle tissue may interact with bone through myokines, which have various physiological on bone metabolism [23].

This study has several limitations that should be mentioned. First, because of its cross-sectional design, these results do not necessarily represent causation. Second, some covariates in the present analysis were self-reported, which may introduce recall bias. Third, the quantitative analysis of BMTs in the NHANES cycles 1999-2000 and 2001-2002 used different assay methods. As a result of different analytic methodology, the BTMs were adjusted in the 2001-2002 cycle. However, it is unlikely that this technical variation may alter the results. Finally, muscle strength which is associated with bone metabolism [23] and mobility limitation [24] was not included as a potential confounder in this study. Despite these limitations, the present results may be generalized to community-dwelling older adults. 

## Conclusions

In community-dwelling older adults, walking speed was significantly correlated with bone turnover markers. Indeed, older men and women with slower walking speeds had consistently higher bone turnover markers levels than those who did not. Moreover, subjects with mobility limitation had evidence of increased bone resorption than their counterparts without. The present findings indicate that older adults with mobility limitation should be screened for osteoporosis and considered at increased risk for fragility fractures. Of interest, it appears that muscle-strengthening exercise may be an effective intervention to attenuate bone resorption in older adults with mobility limitation.

## References

[1] Liu J, Curtis EM, Cooper C, Harvey NC (2019). State of the art in osteoporosis risk assessment and treatment. J Endocrinol Invest.

[2] Maïmoun L, Sultan C (2011). Effects of physical activity on bone remodeling. Metabolism.

[3] Smith C, Tacey A, Mesinovic J (2021). The effects of acute exercise on bone turnover markers in middle-aged and older adults: A systematic review. Bone.

[4] Chen JS, Cameron ID, Cumming RG (2006). Effect of age-related chronic immobility on markers of bone turnover. J Bone Miner Res.

[5] Theiler R, Stähelin HB, Kränzlin M, Tyndall A, Bischoff HA (1999). High bone turnover in the elderly. Arch Phys Med Rehabil.

[6] Bischoff H, Stähelin HB, Vogt P, Friderich P, Vonthein R, Tyndall A, Theiler R (1999). Immobility as a major cause of bone remodeling in residents of a long-stay geriatric ward. Calcif Tissue Int.

[7] Abe Y, Nishimura T, Arima K (2016). Effect of self-reported walking difficulty on bone mass and bone resorption marker in Japanese people aged 40 years and over. J Physiol Anthropol.

[8] (2021). National Health and Nutrition Examination Survey: Plan and Operations. (1999-2010). https://www.cdc.gov/nchs/data/series/sr_01/sr01_056.pdf.

[9] Miller ME, Magaziner J, Marsh AP (2018). Gait speed and mobility disability: revisiting meaningful levels in diverse clinical populations. J Am Geriatr Soc.

[10] Cosman F, de Beur SJ, LeBoff MS, Lewiecki EM, Tanner B, Randall S, Lindsay R (2014). Clinician's guide to prevention and treatment of osteoporosis. Osteoporos Int.

[11] Kitagawa J, Nakahara Y (2008). Associations of daily walking steps with calcaneal ultrasound parameters and a bone resorption marker in elderly Japanese women. J Physiol Anthropol.

[12] Brooke-Wavell K, Jones PR, Hardman AE, Tsuritan Tsuritan, Yamada Y (2001). Commencing, continuing and stopping brisk walking: effects on bone mineral density, quantitative ultrasound of bone and markers of bone metabolism in postmenopausal women. Osteoporos Int.

[13] Yamazaki S, Ichimura S, Iwamoto J, Takeda T, Toyama Y (2004). Effect of walking exercise on bone metabolism in postmenopausal women with osteopenia/osteoporosis. J Bone Miner Metab.

[14] Garnero P, Sornay-Rendu E, Chapuy MC, Delmas PD (1996). Increased bone turnover in late postmenopausal women is a major determinant of osteoporosis. J Bone Miner Res.

[15] Gerdhem P, Ivaska KK, Alatalo SL (2004). Biochemical markers of bone metabolism and prediction of fracture in elderly women. J Bone Miner Res.

[16] Stel VS, Pluijm SM, Deeg DJ, Smit JH, Bouter LM, Lips P (2004). Functional limitations and poor physical performance as independent risk factors for self-reported fractures in older persons. Osteoporos Int.

[17] Barbour KE, Lui LY, McCulloch CE (2016). Trajectories of lower extremity physical performance: effects on fractures and mortality in older women. J Gerontol A Biol Sci Med Sci.

[18] Gombos GC, Bajsz V, Pék E, Schmidt B, Sió E, Molics B, Betlehem J (2016). Direct effects of physical training on markers of bone metabolism and serum sclerostin concentrations in older adults with low bone mass. BMC Musculoskelet Disord.

[19] Beavers KM, Beavers DP, Martin SB (2017). Change in bone mineral density during weight loss with resistance versus aerobic exercise training in older adults. J Gerontol A Biol Sci Med Sci.

[20] Gómez-Cabello A, Ara I, González-Agüero A, Casajús JA, Vicente-Rodríguez G (2012). Effects of training on bone mass in older adults: a systematic review. Sports Med.

[21] Cawthon PM, Manini T, Patel SM (2020). Putative cut-points in sarcopenia components and incident adverse health outcomes: an SDOC analysis. J Am Geriatr Soc.

[22] Grosicki GJ, Travison TG, Zhu H (2020). Application of cut-points for low muscle strength and lean mass in mobility-limited older adults. J Am Geriatr Soc.

[23] Luo Y, Jiang K, He M (2020). Association between grip strength and bone mineral density in general US population of NHANES 2013-2014. Arch Osteoporos.

[24] Jung S, Yabushita N, Kim M (2016). Obesity and muscle weakness as risk factors for mobility limitation in community-dwelling older Japanese women: A two-year follow-up investigation. J Nutr Health Aging.

